# Biomimetic stochastic race model in the subcortical saccadic selection processes: a model of the tecto-basal loops

**DOI:** 10.1186/1471-2202-14-S1-P145

**Published:** 2013-07-08

**Authors:** Charles Thurat, Steve N'Guyen, Benoît Girard

**Affiliations:** 1 ISIR, CNRS-UPMC, Pierre et Marie Curie university, Paris, France; 2 LPPA, CNRS-Collège de France, Paris, France

## 

Gated accumulators, a.k.a race models, have been successfully used to describe neural selection processes, especially in visual selection [1,2]. The elements competing in the visual field feed evidence to counters, up to some selection threshold: the first counter reaching it is selected. Yet, these models do not explain which neurophysiological mechanism could account for the selection threshold itself, as well as how the activity in the accumulators, after threshold crossing, is transformed into a non-ambiguous motor command.

Thus, we propose to create a model of saccade target selection including the Superior Colliculus (SC) and the Basal Ganglia (BG): their reciprocal connectivity has recently been emphasized [5], as well as their implication in visual selection processes [6]. By creating a model linking together recent SC [7] and BG [8] models, we investigate the possibility that selection in the SC results from SC-BG interaction rather than purely lateral inhibitions within the SC and we propose a new role for the Visuo-Motor prelude neurons of the intermediate layers of the SC [4], as noisy evidence integrators for each of the targets presented in the visual input layers. We also propose that the this integrator layer feed the BG, and that the BG disinhibitory feedback modulates the rate of integration in order to stochastically bias the selection process when targets of similar saliences compete. A second feedback loop from the BG to the SC applies direct inhibition to the ouptut of the integration layer to the deepers layers of the SC, and will selectively disinhibit only the winning target and suppress the activity of the distractors or unselected targets, so that the deep motor layer of the SC can produce an unbiaised motor command to the eyeplant.

Our model's architecture offers a mechanism of gluing of the two hemifields for near-vertical saccades, and is able to reliably reproduce selection data and neurons activity profiles gathered in-vivo [3,4], by selecting one target among several distractors of inferior salience as well as one target among several other targets of similar salience. Furthermore, the model provides specific temporal and spatial discrimination predictions that can be tested in-vivo.
